# Advocacy in Action: Leveraging the Power of Patient Voices to Impact Ovarian Cancer Outcomes in Canada

**DOI:** 10.3390/curroncol29020106

**Published:** 2022-02-18

**Authors:** Elisabeth Baugh, Alicia Tone, Talin Boghosian, Alison Ross, Cailey Crawford

**Affiliations:** 1 School of Continuing Studies, University of Toronto, 158 St George St, Toronto, ON M5S 2V8, Canada; 2 Ovarian Cancer Canada, 145 Front St E #205, Toronto, ON M5A 1E3, Canada; atone@ovariancanada.org (A.T.); tboghosian@ovariancanada.org (T.B.); aross@ovariancanada.org (A.R.); ccrawford@ovariancanada.org (C.C.)

**Keywords:** ovarian cancer, patient advocacy, research funding, awareness campaign, OvCAN, patient partners in research

## Abstract

Prior to 1997, ovarian cancer (OC) was a ‘poor target’ for patient advocacy. At that time, there were only three OC researchers in Canada, little information available for women diagnosed, and no community of survivors existed. The Corinne Boyer Fund to advance OC was founded in 1997 (later renamed the National Ovarian Cancer Association (NOCA) and subsequently Ovarian Cancer Canada (OCC)), and a Blueprint for Action was established. NOCA developed training programs for public education, partnered with clinicians and scientists, established a Tissue Banking Network across Canada In 2015, the Ladyballs awareness campaign was launched nationally, giving the community a presence and voice. Strategic planning by the organization put advocacy for research funding as a top priority and, working with patients and researchers across the country, petitioned the government for C$10 million in research funding. In 2019, OCC received the funding. In 2020, the OvCAN project was launched with the aim to improve the outcomes of women diagnosed with OC. In the first three years of OvCAN, a pan-Canadian team of 25 Patient Partners was established, and 41 projects to date on research models, pre-clinical and clinical trials covering a wide spectrum of OC types have been funded.

## 1. Introduction

While the first-ever efforts in advocacy, defined as “one who acts on behalf of another” can be traced back to the 14th century Ming dynasty [1], patient advocacy emerged as a movement in the 1920s as rights for patients became a key role for nurses in their code of ethics and the 1940s, as WWII brought a Citizen Advice Bureau to Great Britain. The breast cancer and HIV/AIDS patient advocacy movements in the 1970s through the 1990s truly identified the characteristics of successful patient empowerment in advocating for policy and healthcare system change. These movements and studies about their success have dominated literature and established best practices for advocacy, setting the stage for a significant shift in health care practice. Patients now demanded a voice in decisions related to their care, and even more broadly, in research decisions, treatment practices and drug access.

**Figure 1:**
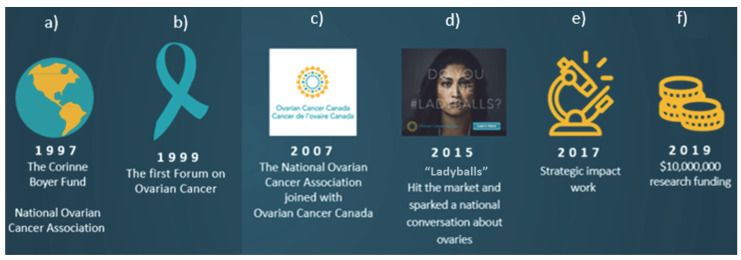
Twenty years of progress (**a**) The Corinne Boyer Fund is established, renamed the National Ovarian Cancer Association; (**b**) the first Forum on Ovarian Cancer; (**c**) the National Ovarian Cancer Association joined with Ovarian Cancer Canada; (**d**) “Ladyballs” national campaign launched, sparking a conversation about ovaries; (**e**) strategic impact work; (**f**) Ovarian Cancer Canada receives C$10,000,000 investment for funding ovarian cancer research.

Ovarian cancer was a new frontier in the late 1990s. Thomas et al. explore a conceptual framework of self-advocacy in women with cancer, and the constructs necessary: informed decision-making, effective communication with health care providers, and connected strength, which all contribute to developing the ability to self-advocate and subsequently the collective role of individuals in an advocacy movement [2]. These were significant building blocks not present for ovarian cancer, making it a ‘poor target’ for patient advocacy [3]. In 1997 in Canada, work began to address this (Figure 1).

## 2. 1997–1999: Early Development of a Canadian Ovarian Cancer Movement

When Corinne Boyer was diagnosed with ovarian cancer in the early 1990s, she was a cancer veteran having already survived melanoma and breast cancer. But the diagnosis of ovarian cancer brought the shocking realization that this cancer was different. There was little or no lay information, no support groups and treatment had not changed in 30 years. There was no screening or early detection tests and with vague signs and symptoms which mimicked more common and benign conditions, most patients were diagnosed in late-stage disease. Standard treatment was primary debulking surgery followed by chemotherapy, a carboplatin cocktail. If one was platinum-resistant and the disease recurred in 6 months, further treatment was palliative. The 5-year mortality rate was 60%. In 1995, Corinne succumbed to her disease. Her husband Patrick Boyer, a prominent politician, set about to address the gaps they had experienced during her care.

These gaps in treatment were significant, but not the only ones. There were only three ovarian cancer researchers in Canada at the time. There was very little funding available for ovarian cancer research and it was not an attractive career choice. Furthermore, given the high mortality rate, and the relatively low incidence of the disease (one in 70 women, or 3100 diagnoses per year in 2021), there was no connected community of survivors to support each other, very few support groups, and very few women surviving or well enough to begin to build a common voice. Unlike breast cancer, there was no community of women living with the disease which could be galvanized into action.

Building a movement takes time and thoughtful deliberate action. In 1997, Patrick Boyer founded the Corinne Boyer Fund to advance ovarian cancer research and to raise awareness of the disease in Canada. In 1999, the name was changed to the National Ovarian Cancer Association (NOCA) (Figure 1a).

In May 1999, NOCA organized Forum ‘99—a 3-day multidisciplinary meeting where 135 scientists, clinicians, policy makers, patients and their families gathered to discuss the challenges and gaps related to building capacity and knowledge about ovarian cancer to move towards improved outcomes (Figure 1b). Based on previous breast cancer and prostate cancer forums, the outcome was a Blueprint for Action identifying several action areas: support, advocacy, research, education and awareness.

## 3. 2000–2005: Building Collective Strength and Clinical Partnerships

In implementing this action plan, the goal of NOCA was to provide resources to support women through the treatment journey—information, support and networking experiences and opportunities to be involved. Training programs were developed for public education—“lunch and learn” sessions, and a regional organization structure was developed to help reach women in every area of Canada. Trained volunteers told other women about the signs and symptoms and fund-raised to support new programs and research. Access to accurate and timely information about the disease to be a participant in your own health care decisions is an initial critical step in becoming an advocate [4]. More than 25 educational symposia and workshops were carried out between 2004 and 2010 with expert content speakers providing those with ovarian cancer with skills in effective communication with their health care providers. For many, this was the first chance to network with other women with ovarian cancer. The opportunities to volunteer, spread the word or participate in fundraising activities further built “connected strength” as women met other women and shared their common experiences. Mok and Martinson referenced the strength that patients draw from each other in sharing, and many of these volunteers reported the support and outreach work was what “kept them going”, using their own experience to help others [3,5].

Partnerships with respected clinicians and scientists were critical to building awareness of the work being done to create engagement and confidence in NOCA. One of the most significant partnerships was with the Society of Gynecologic Oncology of Canada (GOC), the professional association representing the multi-disciplinary clinicians who treat ovarian cancer. NOCA created a position on its Board of Directors for a representative of the GOC, opening the door for regular and ongoing dialogue. Liaison was established with cancer centres as well as primary nurse contacts through the Canadian Association of Nurses in Oncology, who were also granted a board position.

Advocacy was not yet a major thrust; however, there had been an opportunity to test the waters in the early 2000s, when Ontario gynecologic oncologists threatened job action if the government did not negotiate an alternate payment plan for their specialty. This plan would need to address the increased workloads due to distance consulting and an increase in complex surgical procedures. Through a mail campaign, women in Ontario affected by ovarian cancer were provided with a template by NOCA and invited to write to their Member of Provincial Parliament and the provincial health minister, demanding that negotiations begin. It was the first use of this “voice” as a community. And it was successful. A strike was averted and successful negotiations resolved the issue without any gaps in service delivery.

It was always understood by the leadership of NOCA (both staff and board of directors) that research would have to be a central component of the organizational program portfolio; only research would ultimately change outcomes. It was challenging for a small start-up organization to have a significant impact with limited financial resources, but strategic initiatives were identified which would build capacity.

A Tissue Banking Network was established by NOCA with researchers in Montreal, Ottawa and Vancouver in 2002 to ensure ovarian cancer tissue was available for research as needed. Opportunities were identified for matching grant funding with a number of partners to leverage donor funds available and double the impact of the opportunity. In the first year of partnership with the Cancer Research Society, three co-funded grants were planned. The call for projects specifically related to ovarian cancer (the first time this had been done) resulted in more grant applications for ovarian cancer than ever received before. The applications were of such high quality that while NOCA and the Cancer Research Society co-funded three, the Cancer Research Society funded another four grants, bringing the total funded grants for ovarian cancer to 7: 10% of all the cancer research grants funded that year.

Dr. Barbara Vanderhyden, Corinne Boyer Chair in Ovarian Cancer Research (a chair position established in partnership with the University of Ottawa in 2000) had initiated a biannual research conference in partnership with NOCA in 2002 to bring together scientists and clinicians working in ovarian cancer research to discuss and share their expertise. This event galvanized discussion and collaboration within the community and also provided an opportunity for trainees to engage with the best in their field for inspiration and mentorship. Attendance to the conference grew dramatically over the next decade, as the number of researchers in Canada increased as did opportunities for collaboration.

## 4. 2006–2015: Uniting the Voices and Changing the Conversation

By 2006, a strong national organizational presence was in place; however, the existence of a second national ovarian cancer organization, based in Vancouver, was creating division in this community. In her book, “Patient No More” which covers the growth and trajectory of the breast cancer movement in Canada, Sharon Batt (1996) describes the challenges and indeed detrimental effect of a divided patient community on a movement pushing for health care policy change and increased research funding [6]. At times, in-fighting got in the way of a single powerful voice. For the two ovarian cancer groups (NOCA and Ovarian Cancer Canada), there was duplication of services and while Ovarian Cancer Canada was smaller, and largely focused on support, two entities meant inefficiencies and held back the opportunity to build momentum. In 2006, the boards of directors of each group met and made the decision to create one organization. One of the factors in the success of this amalgamation was branding, and the larger NOCA agreed to take on the name of the smaller group, Ovarian Cancer Canada (Figure 1c). Not only were women in the community pleased with this merger, as it meant one comprehensive resource, but the clinicians and researchers also embraced the change as it streamlined all efforts. For donors, it meant a clearer view of the value proposition in one united effort—and clear impact of their gifts.

Following the amalgamation of the two groups in 2007, efforts to increase opportunities to build the community and create cohesion and unity were aided by an increase in support activities, and an increase in engagement of women and their families in fundraising activities. This raised much needed funds and brought groups of people together for the cause, solidifying the community network itself. Awareness of the urgency of changing poor outcomes was a focus and it was clear that much-needed research was the only way survival outcomes would ever change. Over the years, a series of national surveys carried out by Harris Decima, had provided evidence that knowledge of ovarian cancer as the most fatal gynecological cancer was very low, and not shifting significantly over time. Now that there was one community, it was time to galvanize its members and increase public involvement.

In 2015, the Ladyballs campaign to raise awareness of ovarian cancer was launched nationally with a 30 s video spot, a 60 s cinema version and an on-premise film in restaurants, bars and salons. It depicted situations such as business meetings and social gatherings—where women have to make brave decisions. Playing on the analogy of strength related to ‘having balls’, these scenarios were used to remind women that they have balls too (in this case, ovaries), leading to the line, “Do you have the ladyballs to do something about ovarian cancer?” (Figure 1d). Print and digital ads featured women of varying ages and ethnicities to show that the disease does not discriminate. “Show us your ladyballs”—a user-generated activation—was deployed in social, digital and search, allowing the public to display their support for the cause, while PR and community outreach amplified the message. Ovarian Cancer Canada representatives and women living with the disease were also featured in national television and radio news segments. The campaign ran until May 2016. Although controversial, this bold and edgy campaign cut through the noise of a crowded health information marketplace. It was featured in Maclean’s magazine and on the national news, winning seven national and international awards, including a Silver Lion in Cannes in the Health and Wellness marketing category. The disease had made it into mainstream conversation and the organization was poised for the next step. After its creation by Grey Canada, the campaign received more than C$12 million in donated advertising space when Lauren Richards, principal of Toronto media consultancy Pollin8, who herself was diagnosed with ovarian cancer, enlisted Canadian broadcasters, newspapers, magazines and online publishers to donate space and time for the campaign. An unintended effect of the Ladyballs campaign was the impact it had on the mindset and voice of the patient community. One survivor stated that she did not have the Ladyballs to ask for help, she had the Ladyballs to demand action.

In the lead up to the 2015 Federal election, the community was asked to join a “Thunderclap” campaign. This was an online Twitter social media strategy linked to World Ovarian Cancer Awareness Day. The goal was to amplify an important message: “#ovariancancer claims the lives of five Canadian women each day. We demand action from our national leaders RT #elxn42”. A community which had seen itself as the underdog to more prevalent cancers, at last had a presence and a voice.

## 5. 2016–2019: Advocacy Momentum Seated in Sound Business Planning

The development of a new class of drugs, PARP inhibitors, provided a national opportunity for patient advocacy through Ovarian Cancer Canada submissions to The Canadian Agency for Drugs and Technologies in Health (CADTH) pan-Canadian Oncology Drug Review (pCODR) drug review and approval process. The subsequent advocacy for provincial funding to fund these drugs reinforced again the urgency to address the high mortality rate of ovarian cancer. Creating a sense of urgency and running out of time were identified by Lindén (2021) as critical key messages in her study of patient advocacy for access to PARP [2].

In late 2018, a crisis of care erupted in the Prairies. It was announced that two of Saskatchewan’s already few gynecologic oncologists would be leaving the province, leaving a health care gap for women diagnosed with, or at risk of, the disease. Women were concerned, fearful, and had great anxiety about how their care would be impacted. Ovarian Cancer Canada teamed up with local advocates and community members to push for a breakthrough on this doctor shortage issue, including several meetings with Premier Scott Moe and other provincial ministers with health portfolios. In Spring 2019 that sought-after breakthrough became reality when the province announced it was finalizing contracts for the hiring of six permanent gynecologic oncologists, including a new program lead.

Jim Collins, author of “Good To Great” studied hundreds of for-profit companies in developing his model of what makes businesses successful [7]. He also studied non-profit organizations, to help understand the business framework that makes a non-profit “great”, and he outlines these in his monograph “Good to Great for the Social Sectors” [8]. While many of the principles are similar to the for-profit world, Collins pulls apart what is different, what drives an organization forward and demonstrates how to calibrate success without business metrics. While much of what charitable organizations do can be measured, much does not fit a standard model of measurable inputs and outputs. Collins states, “It really doesn’t matter whether you can quantify your results. What matters is that you rigorously assemble evidence, qualitative or quantitative—to track your progress” [8] (p. 7). For Ovarian Cancer Canada, the evidence from these early successes was clear—the advocacy voice was growing and the community was mobilizing.

Examining the steps in successful advocacy techniques which built the ovarian cancer movement in Canada, one must consider the strategic business planning of the patient organization over 20 years. This provided a sound framework for the work of building a community, educating it, activating it, identifying the challenges to changing outcomes, assessing ability to overcome these, setting the plan in place, and then achieving it.

Ovarian Cancer Canada’s plan exemplified Collins’ Hedgehog Concept—his model for success: finding the intersection of what you are passionate about, what drives your resource engine, and what you can be best in the world at. “You begin with passion, then you refine passion with a rigorous assessment of what you can best contribute to the communities you touch. Then you create a way to tie your resource engine directly to the other two” [7] (p. 19). This combination builds results which in turn attract resources, building a stronger organization, attracting more resources. This excites a community that wants to be part of the change. “Success breeds support and commitment” [8] (p. 24).

As Ovarian Cancer Canada demonstrated success in building a community, setting goals, achieving them and bringing an advocacy voice to new tables, the organization’s leadership wanted to go further to explicitly outline linkages between initiatives and the results it sought to achieve. If its goal was to “affect changes in ovarian cancer that would save women’s lives”, it wanted to test out its working assumptions between the actions the organization took and the results it expected. Developing a theory of change would provide the rigor that would instill discipline in its activities and confidence from the community it served [9].

Through consultation with the clinical and research communities, Ovarian Cancer Canada was certain that its ultimate goal to save women’s lives would only be achieved with an increase in research investment. The evidence connecting increased research investment to improved cancer outcomes was clear. For example, the 10-year federal research investment between 2005–2015 in breast and prostate research funding (both higher incidence cancers) was C$249.3 M and C$102.7 M, respectively, with 5-year survival rates of 87% and 95%. This compared to a dismal investment of C$8.6 M in ovarian cancer and survival rates of 44% which had not changed in 50 years.

Strategic planning by the organization made advocacy for increased research funding a top priority, and again, working with researchers across the country, a plan was developed (Figure 1e). Over and over, women reported they had run out of treatment options. What would it take to bring new treatments to the market which would extend the length and quality of life? Would a C$10 million investment in research be a good starting point? Ovarian Cancer Canada consulted with members of The Ovarian Cancer Research Consortium consisting of 250+ researchers from across Canada, including 70+ clinical members specializing in the diagnosis and treatment of gynecologic cancers (gynecologic oncologists, medical oncologists, pathologists, radiation oncologists) and representation from academic institutions in eight provinces. Consortium members confirmed C$10 million would make a difference and worked with Ovarian Cancer Canada to develop a three-pronged research strategy named OvCAN and advocacy for research funding began in earnest.

Linking advocacy theory and business success, there were a number of factors in place which contributed to this effective advocacy strategy:

- A united community with one voice which included experts—women living with the disease and researchers;
- A credible and solid plan with consistent key messages;
- Understanding of the ruling party priorities—in this case Justin Trudeau’s Liberal government, which had a feminist platform and more female members of parliament than ever before—and how to mesh the strategy with those priorities;
- An ongoing, focused plan to engage policy makers, build partnerships and leverage key opportunities;
- Brand reputation—a demonstration that the organization was capable and equipped to carry out the proposed plan.

Changes in the external environment also supported the timing of this movement:

- Technology and access to information—use of social media had exploded since 2000;
- Understanding of ovarian cancer had also developed significantly—from the assumption that it was one disease to evidence that it consists of several distinct diseases that differ in many aspects, including histology, cell/tissue of origin, molecular alterations, response to treatment, and patient prognosis which demanded increased targeted research.

An external agency, Hill and Knowlton was hired to develop the political strategy, capitalizing on the strengths of the organization: a solid track record, an engaged patient community and support from the research community. Working with Hill and Knowlton, key thought leaders were identified in all political parties. Stakeholders across the country were alerted to help identify politicians who might be champions for this proposal and worked with staff, the board of directors, and researchers to leverage any personal contacts they might have to increase national support. Key staff in the offices of members of parliament were also engaged as champions, because of personal experience with the disease or as women’s health advocates.

### Momentum and Persistence

From 2016–2018, more than 3000 Canadians wrote letters to their MPs, and 12,703 signed a petition calling for an investment of an additional C$10 million in funding for ovarian cancer research which was presented in the House of Commons on 24 February 2017. Their message was a personal one- their experience, the lack of treatments available, and the urgent need for funding.

Ovarian Cancer Canada trained 50 volunteer advocates and each “advocacy team” was strategically formed with a staff person or member of the board of directors, a woman living with ovarian cancer or a family member, and a researcher. The success of these advocacy teams was rooted in the hybridization of expert research knowledge and lay experience. Each member of the advocacy team had a key role, bringing specific experience and knowledge to the meeting. These advocates travelled to Ottawa 3 years in a row for “Days on the Hill” which included meetings with MPs, senators and key staff members in various ministries, and all-party receptions. There were also meetings in local ridings across the country with MPs consistently driving the message home: Ovarian cancer had been overlooked and underfunded, outcomes had not changed in 50 years, and only a research investment could change this. The advocacy dialogue shifted over 4 years as engagement built and champions were identified who would help move the “ask” forward.

Initially, building a basic understanding of the nature of ovarian cancer was required as a low level of awareness about the disease was evident. In a first meeting with the head of the Women’s Caucus, she stated she was well aware of the dangers of the disease and that was why she always had her annual pap screening. The Ovarian Cancer Canada team congratulated her for her diligent heath practice, however it noted test only screening for cervical cancer, and in fact there was no screening test for ovarian cancer.

The next stage was explaining why funding for general cancer research was not benefiting ovarian cancer outcomes, as the unique features of ovarian cancer meant that it did not benefit from the transfer of knowledge from findings about other cancers. Next it was necessary to explain why existing federal research funding programs were not adequate or appropriate for ovarian cancer. For example, ovarian cancer was not rare enough to qualify for funding for research into rare cancers, and not fatal enough to fit into high mortality criteria. Yet five Canadian women were dying every day of the disease, women at the centre of family life with careers and children losing years of life and the opportunity to contribute to the fabric of the Canadian economy. A critical step here was to get confirmation from the funding bodies themselves that this was a valid assessment. It was a marathon, not a sprint, to the finish line.

Over the course of four years, Ovarian Cancer Canada held over 100 meetings with MPs and Senators, presented to the pre-budget hearings to the House of Commons Standing Committee on Finance (2017) and made two Budget submissions (2018 and 2019). A cabinet shuffle often meant backtracking and building new relationships as staff and ministers changed. The organization leaned upon its champions, MPs and key staff who were known to be sympathetic to the cause and would help move the request forward. A key factor in the success over the 4-year advocacy push was reiterated by politicians met by the Ovarian Cancer Canada team: what was asked of the community never changed, the plan never changed, and the team was unwavering in their goal.

## 6. 2019–2021: Achieving C$10 M Ovarian Cancer Research Federal Investment

In March 2019, an election year, the federal government approved funding of C$10 million for ovarian cancer research to Ovarian Cancer Canada (Figure 1f). While funding was approved, there were challenges in the release of the funds. In January 2020, the OvCAN project was finally launched, a three-pronged approach with a clear single aim over 5 years: to improve the outcomes of women diagnosed with ovarian cancer. To address this aim, the following three ovarian cancer research objectives made up the plan:

(1) Develop new research models to bridge the gap between discovery and validation of new treatments by funding the researchers and mechanisms that can optimize existing and new models for pre-clinical trials and high-throughput assessment (including human ovarian cancer cell lines, ex vivo models/organoids, and in vivo models);
(2) Identify and prioritize novel treatments to be tested in the best preclinical research models by funding research of hit-to-lead targets and novel therapeutic approaches;
(3) Advance clinical trials by stratifying ovarian cancer patients to experimental treatments based on the defining features of their tumour (i.e., using a personalized medicine approach).

A multi-disciplinary, pan-Canadian Governing Council was established to oversee the project with representation from the scientific, clinical and patient communities, as well as leadership from Ovarian Cancer Canada. Over the next 12 months, the C$10 million funding was further leveraged through granting partnerships with IRICoR and the Cancer Research Society to C$11.75 million. Furthermore, patient advocates in Nova Scotia and Saskatchewan secured a further C$1 million from each province to support increasing research capacity within the OvCAN parameters in those provinces. The plan was to roll this funding push out across the country, however the onset of the pandemic in March 2020 put a halt to plans temporarily. Nonetheless, already, in 4 short months, the original C$10 M had been leveraged to C$13.75 M.

Ovarian Cancer Canada added a fourth priority to OvCAN to ensure that the patient voice was consistently heard at the table as funding decisions were made. Literature reflects a growing view that patients should be more actively involved as participants in the planning process for health research, providing important forms of expertise that are vital to the development of new therapies [10]. Building on best practice examples from the American Association for Cancer Research, the Canadian Cancer Research Alliance and BioCan Rx, Ovarian Cancer Canada developed a Patient Partners in Research (PPIR) Program which trained women living with ovarian cancer to participate in grant decisions, providing them with the adequate scientific understanding and knowledge of the grant process so they could effectively add their experience and opinions to the decision-making process. The PPIR team is the largest group of its kind in Canada, consisting of 25 women from seven provinces. PPIR team members are involved in many activities including participation in monthly educational meetings with expert speakers, serving as patient reviewers on grant review panels for pre-clinical studies and clinical trials, participating as embedded team members in OvCAN-funded clinical trials, interactive sessions with ovarian cancer research trainees and lab staff and serving as speakers and panelists at the virtual Ovarian Cancer Canada symposia. The program has been so successful that grant partners have reported they are expanding this model into their work with other partners.

## 7. What’s Next: Beyond OvCAN—A Multi-Pronged National Approach for Improving Ovarian Cancer Outcomes in Canada

Ovarian Cancer Canada has made great progress in the first 3 years of the OvCAN 5-year initiative, by strengthening the collaborative network of ovarian cancer researchers in Canada, establishing a pan-Canadian team of 25 Patient Partners in Research, and funding a combined 41 projects to date on state-of-the-art research models, pre-clinical studies, and personalized clinical trials covering a wide spectrum of ovarian cancer types. At the same time, the organization’s work with patients and clinicians has revealed regional variations and systemic gaps in genetic testing, treatment, clinical trial access, risk-reducing surgery and after-care, and information resources. There is much more work to be done, and no time to wait. The advocacy work has not finished with the research funding. Ovarian Cancer Canada is developing “Beyond OvCAN” a comprehensive strategy that will build on the current momentum in OvCAN and other Ovarian Cancer Canada research initiatives to optimize outcomes along the full ovarian cancer continuum (prevention, diagnosis, treatment, supportive care).

Other Ovarian Cancer Canada research initiatives include:

- The Every Woman Study: Canadian Edition, a national survey of more than 550 Canadian women living with ovarian cancer which identified systemic gaps in care and regional variations in care including genetic testing, treatments, and clinical trial participation.
- The State of the Nation clinical audit, a multi-province study that will report on incidence, prevalence, staging, and mortality to create an “atlas” of ovarian cancer in select provinces (British Columbia, Saskatchewan, Manitoba, Ontario, and Nova Scotia). This atlas seeks to map meaningful indicators of access and quality of care against these data, where possible.
- Prevention Research: to improve outcomes it is necessary to maximize ovarian cancer prevention opportunities. Ovarian Cancer Canada is conducting surveys, interviews, and focus groups with previvors (those with genetic mutations that increase their risk for developing ovarian cancer), genetic counselors, and surgeons who perform risk-reducing bilateral salpingo-oophorectomy and opportunistic salpingectomy surgeries. Work to date already reveals regional variations and systemic gaps regarding access to genetic counselling, genetic testing, and risk-reducing surgery for identified previvors, and uptake of opportunistic salpingectomy for those at average risk for ovarian cancer.

All ‘Beyond OvCAN’ endeavors will be rooted in two philosophies:

(1) Patient-centricity and expression will be inherent to all research activities. This is to ensure that all work is representative of a broad patient experience, accounting for factors such as ovarian cancer type, geography, age, urban or rural location, race, ethnicity, sexuality, gender and socioeconomic status.
(2) Health inequities are the result of the different, systematic, and preventable social conditions that people face, collectively referred to as social determinants of health [11]. To promote health equity, commitments and investments must be made locally, nationally, and globally to improve social conditions. The values of equity, diversity, and inclusion will be prioritized to address historic and systemic inequities in clinical research and care.

Results of this work will create a compelling evidence base from which Ovarian Cancer Canada will identify priorities, mobilize stakeholders, and advocate for changes in clinical policy and practice to ensure all Canadians have the best possible care regardless of where they live.

## 8. Conclusions

As one reflects on the history of patient advocacy in the past 50 years, it is possible to identify moments of change or breakthrough that subsequently led to a movement responsible for changing the status quo. Author Robert Glazer (2018) says “Truly launching a movement requires sustained action around a deep-rooted purpose…” [12]. The success of the ovarian cancer advocacy movement is seated in a thoughtful, deliberate, multi-faceted business-driven plan executed by Ovarian Cancer Canada which, over 20 years, mobilized a community which had been silent and forgotten, galvanizing the patients, clinicians and researchers around a common goal with measurable positive outcomes.

## Data Availability

No new data were created or analyzed in this study. Data sharing is not applicable to this article.
